# 2-Amino­pyridinium picrate

**DOI:** 10.1107/S1600536810024220

**Published:** 2010-06-26

**Authors:** M. S. Sivaramkumar, R. Velmurugan, M. Sekar, P. Ramesh, M. N. Ponnuswamy

**Affiliations:** a Post Graduate and Research Department of Chemistry, Kongunadu College of Arts and Science, Coimbatore 641 029, India; b Post Graduate and Research Department of Chemistry, Sri Ramakrishna Mission Vidyalaya College of Arts and Science, Coimbatore 641 020, India; c Centre of Advanced Study in Crystallography and Biophysics, University of Madras, Guindy Campus, Chennai 600 025, India

## Abstract

In the title compound, C_5_H_7_N_2_^+^·C_6_H_2_N_3_O_7_^−^, there are two crystallographically independent cations and anions (*A* and *B*) in the asymmetric unit. In both picrate anions, one of the nitro groups lies in the plane of the benzene ring [r.m.s. deviations = 0.014 (2) and 0.014 (2) Å for anions *A* and *B*, respectively] and the other two are twisted away by 39.0 (2) and 18.8 (2)° in *A*, and 18.2 (1) and 2.5 (2)° in *B*. In the crystal, the cations and anions are linked by inter­molecular N—H⋯O and C—H⋯O hydrogen bonds, forming a two-dimensional network.

## Related literature

For general background to picrate complexes, see: In *et al.* (1997 ▶); Zaderenko *et al.* (1997 ▶). 
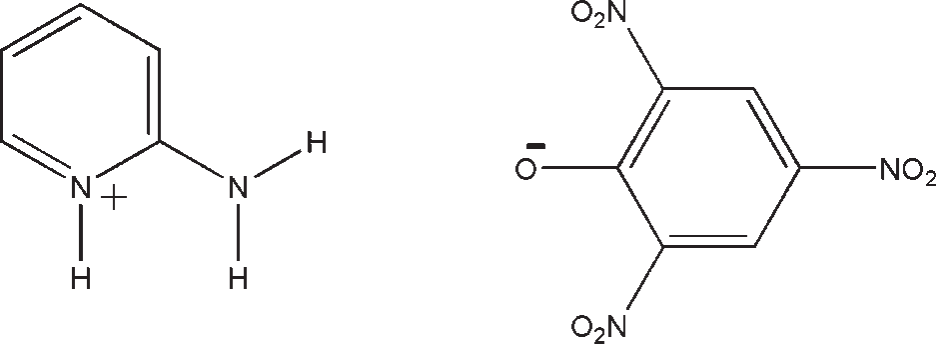


## Experimental

### 

#### Crystal data

C_5_H_7_N_2_^+^·C_6_H_2_N_3_O_7_^−^
*M* *_r_* = 323.23
Triclinic, 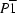

*a* = 11.2543 (4) Å
*b* = 11.6588 (5) Å
*c* = 12.9883 (5) Å
α = 114.641 (4)°
β = 100.204 (3)°
γ = 103.928 (3)°
*V* = 1427.16 (12) Å^3^
*Z* = 4
Mo *K*α radiation
μ = 0.13 mm^−1^
*T* = 110 K
0.20 × 0.17 × 0.15 mm

#### Data collection

Bruker SMART APEXII area-detector diffractometer
Absorption correction: multi-scan (*SADABS*; Bruker, 2008 ▶) *T*_min_ = 0.975, *T*_max_ = 0.981
12905 measured reflections
6555 independent reflections
3011 reflections with *I* > 2σ(*I*)
*R*_int_ = 0.020

#### Refinement

*R*[*F* ^2^ > 2σ(*F* ^2^)] = 0.054
*wR*(*F* ^2^) = 0.179
*S* = 0.90
6555 reflections
438 parameters
1 restraint
H atoms treated by a mixture of independent and constrained refinement
Δρ_max_ = 0.27 e Å^−3^
Δρ_min_ = −0.21 e Å^−3^

### 

Data collection: *APEX2* (Bruker, 2008 ▶); cell refinement: *SAINT* (Bruker, 2008 ▶); data reduction: *SAINT*; program(s) used to solve structure: *SHELXS97* (Sheldrick, 2008 ▶); program(s) used to refine structure: *SHELXL97* (Sheldrick, 2008 ▶); molecular graphics: *ORTEP-3* (Farrugia, 1997 ▶); software used to prepare material for publication: *SHELXL97* and *PLATON* (Spek, 2009 ▶).

## Supplementary Material

Crystal structure: contains datablocks global, I. DOI: 10.1107/S1600536810024220/sj5016sup1.cif

Structure factors: contains datablocks I. DOI: 10.1107/S1600536810024220/sj5016Isup2.hkl

Additional supplementary materials:  crystallographic information; 3D view; checkCIF report

## Figures and Tables

**Table 1:** Hydrogen-bond geometry (Å, °)

*D*—H⋯*A*	*D*—H	H⋯*A*	*D*⋯*A*	*D*—H⋯*A*
N1*B*—H1*B*⋯O1*B*	0.96 (3)	1.79 (3)	2.681 (3)	152 (2)
N1*B*—H1*B*⋯O7*B*	0.96 (3)	2.36 (3)	3.035 (3)	127 (2)
N7*B*—H7*D*⋯O1*B*	0.84 (3)	2.04 (3)	2.784 (3)	148 (3)
N7*B*—H7*D*⋯O2*B*	0.84 (3)	2.47 (3)	3.165 (3)	141 (2)
N1*A*—H1*A*⋯O1*A*	0.87 (3)	1.97 (3)	2.726 (2)	145 (2)
N1*A*—H1*A*⋯O7*A*	0.87 (3)	2.34 (3)	3.031 (3)	137 (2)
N7*A*—H7*B*⋯O2*A*	1.05 (3)	2.40 (3)	3.329 (3)	147 (2)
C10*A*—H10*A*⋯O4*B*^i^	0.95	2.57	3.438 (3)	153
N7*A*—H7*A*⋯O2*B*^ii^	0.87 (3)	2.24 (3)	3.029 (3)	152 (3)
N7*B*—H7*C*⋯O2*A*^ii^	0.72 (3)	2.49 (3)	3.137 (3)	149 (3)

Symmetry codes: (i) 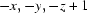; (ii) 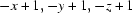.

**Fig. 1.**
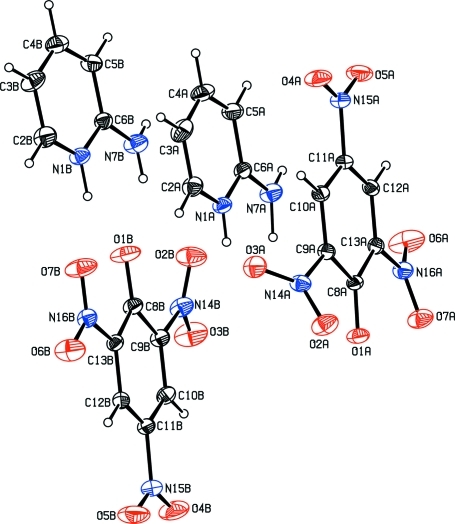
The asymmetric unit of the title compound. Displacement ellipsoids are drawn at the 50% probability level.

**Fig. 2.**
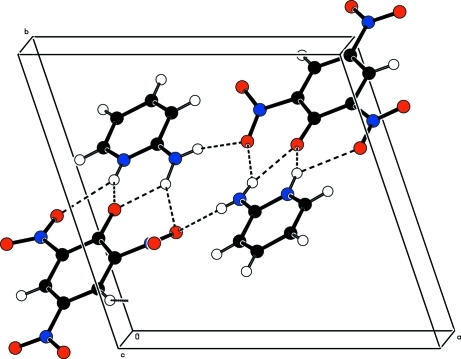
The crystal packing of the title compound, viewed down the c axis.

## supplementary crystallographic information

### Comment

2,4,6-Trinitro phenol, also called picric acid, was primarily used to manufacture explosives and dyes. Picric acid forms molecular charge transfer complexes with aromatic compounds through electrostatic or hydrogen bonding interactions (In *et al.*, 1997; Zaderenko *et al.*, 1997). We report here the crystal structure of the title salt to understand its molecular conformation and packing mode.

There are two crystallographically independent cations and anions (A & B) in the asymmetric unit. Both the pyridinium rings of the cation (Figure 1) are planar (r.m.s. deviations 0.004 (4) and 0.002 (3) Å). In the picrate anion, the keto O atom lies in the plane of the benzene ring [-0.010 (2) Å] in molecule A whereas it deviates by -0.076 (2)Å in molecule B. The C8A—O1A [1.252 (2) Å] and C8B—O1B [1.237 (3) Å] bonds assume partial double bond character. The C8A—C9A (1.446 (3) Å), C8B—C9B (1.450 (3) Å), C8A—C13A (1.436 (3) Å) and C8B—C13B (1.441 (3) Å) bond distances are longer than the normal bond lengths in a benzene ring. In both the anions , one of the nitro groups of the picrate lies in the plane of the benzene ring while the other two are twisted away by 39.0 (2)° [N14A/O2A/O3A] & 18.8 (2)° [N16A/O6A/O7A] for molecule A and 18.2 (1)° [N14B/O2B/O3B] & 2.5 (2)° [N16B/O6B/O7B] for molecule B.

In the crystal, the cations and anions are linked *via* intermolecular N—H···O and C—H···O hydrogen bonds (Table 1), which form a two dimensional network (Figure 2).

### Experimental

2-Amino pyridinium picrate was prepared from a methanol solution containing equimolar amounts of picric acid and 2-amino pyridine. Single crystals suitable for X-ray analysis are obtained by repeated recrystallization of the salt from pure methanol.

### Refinement

The N-bound H atom was located in a difference map and refined isotrophically. C-bound H atoms were positioned geometrically (C–H = 0.93–0.96 Å) and allowed to ride on their parent atoms, with *U*_iso_(H) = 1.2*U*_eq_(C) for all H atoms. A search for solvent-accessible voids in the crystal structure using *PLATON* shows a potential solvent volume of 114.0 Å^3^. However, this procedure showed no electron density in the voids. This indicates that the crystal lost nearly all of its solvent of crystallization by the time it was used for data collection, without collapse of the structure.

### Crystal data

**Table:**

C_5_H_7_N_2_^+^·C_6_H_2_N_3_O_7_^−^	*Z* = 4
*M**_r_* = 323.23	*F*(000) = 664
Triclinic, *P*1	*D*_x_ = 1.504 Mg m^−^^3^
Hall symbol: -P 1	Mo *K*α radiation, λ = 0.71073 Å
*a* = 11.2543 (4) Å	Cell parameters from 1241 reflections
*b* = 11.6588 (5) Å	θ = 2.9–29.2°
*c* = 12.9883 (5) Å	µ = 0.13 mm^−^^1^
α = 114.641 (4)°	*T* = 110 K
β = 100.204 (3)°	Block, colourless
γ = 103.928 (3)°	0.20 × 0.17 × 0.15 mm
*V* = 1427.16 (12) Å^3^	

### Data collection

**Table:**

Bruker SMART APEXII area-detector diffractometer	6555 independent reflections
Radiation source: fine-focus sealed tube	3011 reflections with *I* > 2σ(*I*)
graphite	*R*_int_ = 0.020
ω and φ scans	θ_max_ = 29.2°, θ_min_ = 2.9°
Absorption correction: multi-scan (*SADABS*; Bruker, 2008)	*h* = −14→14
*T*_min_ = 0.975, *T*_max_ = 0.981	*k* = −13→15
12905 measured reflections	*l* = −17→16

### Refinement

**Table:**

Refinement on *F*^2^	Primary atom site location: structure-invariant direct methods
Least-squares matrix: full	Secondary atom site location: difference Fourier map
*R*[*F*^2^ > 2σ(*F*^2^)] = 0.054	Hydrogen site location: inferred from neighbouring sites
*wR*(*F*^2^) = 0.179	H atoms treated by a mixture of independent and constrained refinement
*S* = 0.90	*w* = 1/[σ^2^(*F*_o_^2^) + (0.0994*P*)^2^] where *P* = (*F*_o_^2^ + 2*F*_c_^2^)/3
6555 reflections	(Δ/σ)_max_ = 0.003
438 parameters	Δρ_max_ = 0.27 e Å^−^^3^
1 restraint	Δρ_min_ = −0.21 e Å^−^^3^

### Special details

**Table:**

Geometry. All e.s.d.'s (except the e.s.d. in the dihedral angle between two l.s. planes) are estimated using the full covariance matrix. The cell e.s.d.'s are taken into account individually in the estimation of e.s.d.'s in distances, angles and torsion angles; correlations between e.s.d.'s in cell parameters are only used when they are defined by crystal symmetry. An approximate (isotropic) treatment of cell e.s.d.'s is used for estimating e.s.d.'s involving l.s. planes.
Refinement. Refinement of *F*^2^ against ALL reflections. The weighted *R*-factor *wR* and goodness of fit *S* are based on *F*^2^, conventional *R*-factors *R* are based on *F*, with *F* set to zero for negative *F*^2^. The threshold expression of *F*^2^ > σ(*F*^2^) is used only for calculating *R*-factors(gt) *etc*. and is not relevant to the choice of reflections for refinement. *R*-factors based on *F*^2^ are statistically about twice as large as those based on *F*, and *R*- factors based on ALL data will be even larger.

### Fractional atomic coordinates and isotropic or equivalent isotropic displacement parameters (Å^2^)

**Table:**

	*x*	*y*	*z*	*U*_iso_*/*U*_eq_	
O1A	0.09943 (15)	0.43819 (16)	0.43463 (15)	0.0536 (5)	
O1B	0.23913 (17)	0.3169 (2)	0.1731 (2)	0.0813 (7)	
O2A	0.27377 (16)	0.3677 (2)	0.54622 (19)	0.0748 (6)	
O2B	0.40164 (17)	0.3168 (2)	0.3449 (2)	0.0836 (7)	
O3A	0.20270 (17)	0.33859 (19)	0.67855 (17)	0.0710 (6)	
O3B	0.36036 (19)	0.1314 (2)	0.3494 (2)	0.0942 (8)	
O4A	−0.20594 (19)	−0.04375 (18)	0.48852 (19)	0.0745 (6)	
O4B	−0.0709 (2)	−0.1479 (2)	0.2427 (2)	0.0963 (8)	
O5A	−0.35954 (18)	−0.03632 (19)	0.36907 (19)	0.0752 (6)	
O5B	−0.2227 (2)	−0.1377 (2)	0.1262 (2)	0.1038 (9)	
O6A	−0.28300 (19)	0.3105 (3)	0.2612 (2)	0.1067 (9)	
O6B	−0.14349 (18)	0.1761 (2)	−0.0134 (2)	0.0871 (7)	
O7A	−0.09658 (17)	0.40685 (19)	0.26526 (17)	0.0671 (6)	
O7B	0.03844 (19)	0.3203 (2)	0.0375 (2)	0.0870 (7)	
N1A	0.17461 (19)	0.6011 (2)	0.33902 (18)	0.0435 (5)	
H1A	0.124 (2)	0.534 (3)	0.342 (2)	0.061 (8)*	
N1B	0.31598 (19)	0.4800 (2)	0.08244 (18)	0.0478 (5)	
H1B	0.265 (2)	0.409 (3)	0.093 (2)	0.070 (8)*	
C2A	0.1199 (3)	0.6384 (3)	0.2628 (2)	0.0538 (7)	
H2A	0.0306	0.5945	0.2190	0.065*	
C2B	0.2668 (3)	0.5098 (3)	−0.0024 (3)	0.0631 (8)	
H2B	0.1791	0.4623	−0.0508	0.076*	
C3A	0.1918 (3)	0.7381 (3)	0.2486 (3)	0.0637 (8)	
H3A	0.1542	0.7642	0.1944	0.076*	
C3B	0.3409 (3)	0.6061 (3)	−0.0190 (3)	0.0681 (8)	
H3B	0.3069	0.6277	−0.0781	0.082*	
C4A	0.3221 (3)	0.8020 (3)	0.3147 (3)	0.0616 (8)	
H4A	0.3736	0.8727	0.3057	0.074*	
C4B	0.4689 (3)	0.6726 (3)	0.0531 (3)	0.0621 (8)	
H4B	0.5230	0.7404	0.0427	0.075*	
C5A	0.3761 (3)	0.7650 (2)	0.3913 (2)	0.0556 (7)	
H5A	0.4650	0.8096	0.4364	0.067*	
C5B	0.5183 (2)	0.6432 (2)	0.1379 (2)	0.0527 (7)	
H5B	0.6059	0.6905	0.1865	0.063*	
C6A	0.2995 (2)	0.6592 (2)	0.4042 (2)	0.0417 (6)	
C6B	0.4402 (2)	0.5433 (2)	0.1535 (2)	0.0425 (6)	
N7A	0.3450 (2)	0.6145 (2)	0.4761 (2)	0.0557 (6)	
H7A	0.427 (3)	0.650 (3)	0.514 (3)	0.102 (8)*	
H7B	0.287 (3)	0.528 (3)	0.474 (3)	0.102 (8)*	
N7B	0.4794 (3)	0.5063 (3)	0.2321 (2)	0.0626 (7)	
H7C	0.547 (3)	0.539 (3)	0.265 (3)	0.060 (10)*	
H7D	0.423 (3)	0.450 (3)	0.239 (3)	0.070 (10)*	
C8A	0.0194 (2)	0.3402 (2)	0.43135 (19)	0.0361 (5)	
C8B	0.1588 (2)	0.2278 (2)	0.1784 (2)	0.0453 (6)	
C9A	0.0536 (2)	0.2783 (2)	0.5026 (2)	0.0386 (5)	
C9B	0.1927 (2)	0.1623 (2)	0.2467 (2)	0.0442 (6)	
C10A	−0.0308 (2)	0.1731 (2)	0.5044 (2)	0.0411 (6)	
H10A	−0.0032	0.1371	0.5539	0.049*	
C10B	0.1087 (2)	0.0598 (2)	0.2493 (2)	0.0451 (6)	
H10B	0.1373	0.0203	0.2951	0.054*	
C11A	−0.1580 (2)	0.1204 (2)	0.4318 (2)	0.0385 (5)	
C11B	−0.0197 (2)	0.0135 (2)	0.1839 (2)	0.0437 (6)	
C12A	−0.2003 (2)	0.1743 (2)	0.3635 (2)	0.0389 (5)	
H12A	−0.2883	0.1384	0.3164	0.047*	
C12B	−0.0629 (2)	0.0708 (2)	0.1180 (2)	0.0397 (6)	
H12B	−0.1517	0.0384	0.0742	0.048*	
C13A	−0.1151 (2)	0.2806 (2)	0.36324 (19)	0.0376 (5)	
C13B	0.0225 (2)	0.1740 (2)	0.1161 (2)	0.0379 (5)	
N14A	0.18554 (19)	0.33227 (19)	0.58082 (19)	0.0484 (5)	
N14B	0.3273 (2)	0.2071 (2)	0.3173 (2)	0.0579 (6)	
N15A	−0.2477 (2)	0.00586 (19)	0.4296 (2)	0.0524 (6)	
N15B	−0.1106 (2)	−0.0986 (2)	0.1844 (2)	0.0608 (6)	
N16A	−0.1678 (2)	0.3358 (2)	0.29156 (18)	0.0493 (5)	
N16B	−0.0302 (2)	0.2282 (2)	0.04259 (18)	0.0481 (5)	

### Atomic displacement parameters (Å^2^)

**Table:**

	*U*^11^	*U*^22^	*U*^33^	*U*^12^	*U*^13^	*U*^23^
O1A	0.0453 (9)	0.0539 (10)	0.0624 (12)	0.0011 (8)	0.0076 (8)	0.0413 (9)
O1B	0.0462 (11)	0.1026 (15)	0.1073 (17)	−0.0049 (10)	0.0033 (10)	0.0862 (14)
O2A	0.0380 (10)	0.1079 (16)	0.0862 (15)	0.0112 (10)	0.0115 (10)	0.0646 (13)
O2B	0.0465 (11)	0.0889 (15)	0.1088 (18)	0.0002 (9)	−0.0047 (11)	0.0659 (14)
O3A	0.0661 (12)	0.0843 (14)	0.0561 (13)	0.0079 (10)	−0.0046 (10)	0.0474 (11)
O3B	0.0629 (13)	0.1237 (19)	0.133 (2)	0.0345 (12)	0.0134 (13)	0.1004 (17)
O4A	0.0800 (14)	0.0627 (12)	0.0970 (16)	0.0110 (10)	0.0246 (12)	0.0615 (12)
O4B	0.1063 (18)	0.0910 (16)	0.115 (2)	0.0131 (13)	0.0293 (15)	0.0845 (16)
O5A	0.0518 (12)	0.0658 (13)	0.0883 (16)	−0.0106 (9)	0.0091 (11)	0.0419 (11)
O5B	0.0642 (14)	0.0994 (17)	0.125 (2)	−0.0206 (12)	0.0024 (14)	0.0711 (16)
O6A	0.0481 (13)	0.155 (2)	0.149 (2)	0.0152 (13)	−0.0001 (13)	0.125 (2)
O6B	0.0508 (12)	0.1075 (17)	0.1051 (18)	0.0075 (11)	−0.0084 (11)	0.0770 (15)
O7A	0.0610 (12)	0.0790 (14)	0.0779 (14)	0.0147 (10)	0.0102 (10)	0.0622 (12)
O7B	0.0596 (12)	0.1074 (17)	0.1231 (19)	0.0117 (11)	0.0115 (12)	0.0975 (16)
N1A	0.0437 (12)	0.0441 (12)	0.0469 (13)	0.0100 (10)	0.0118 (10)	0.0295 (10)
N1B	0.0379 (11)	0.0573 (13)	0.0522 (13)	0.0089 (10)	0.0078 (10)	0.0366 (11)
C2A	0.0578 (16)	0.0597 (16)	0.0533 (17)	0.0205 (13)	0.0128 (13)	0.0374 (14)
C2B	0.0525 (16)	0.078 (2)	0.0618 (19)	0.0157 (14)	0.0065 (14)	0.0446 (16)
C3A	0.082 (2)	0.077 (2)	0.0677 (19)	0.0430 (17)	0.0314 (17)	0.0548 (17)
C3B	0.074 (2)	0.083 (2)	0.077 (2)	0.0290 (17)	0.0280 (17)	0.0624 (19)
C4A	0.073 (2)	0.0548 (17)	0.081 (2)	0.0231 (15)	0.0380 (17)	0.0486 (16)
C4B	0.0669 (19)	0.0553 (17)	0.076 (2)	0.0164 (14)	0.0293 (16)	0.0416 (16)
C5A	0.0527 (16)	0.0488 (16)	0.0662 (19)	0.0100 (12)	0.0244 (14)	0.0306 (14)
C5B	0.0447 (14)	0.0481 (15)	0.0608 (18)	0.0077 (11)	0.0136 (13)	0.0279 (13)
C6A	0.0453 (14)	0.0408 (14)	0.0435 (14)	0.0143 (11)	0.0183 (12)	0.0230 (11)
C6B	0.0379 (13)	0.0460 (14)	0.0438 (15)	0.0119 (11)	0.0133 (11)	0.0231 (12)
N7A	0.0419 (13)	0.0706 (16)	0.0612 (16)	0.0119 (12)	0.0070 (11)	0.0453 (13)
N7B	0.0372 (14)	0.0818 (19)	0.0681 (18)	0.0050 (13)	0.0020 (13)	0.0498 (15)
C8A	0.0390 (12)	0.0337 (13)	0.0361 (13)	0.0073 (10)	0.0102 (10)	0.0210 (10)
C8B	0.0393 (13)	0.0530 (15)	0.0489 (15)	0.0108 (11)	0.0113 (11)	0.0332 (13)
C9A	0.0371 (12)	0.0378 (13)	0.0385 (13)	0.0090 (10)	0.0062 (10)	0.0206 (11)
C9B	0.0371 (13)	0.0518 (15)	0.0475 (15)	0.0125 (11)	0.0105 (11)	0.0301 (12)
C10A	0.0508 (14)	0.0338 (13)	0.0418 (14)	0.0122 (10)	0.0128 (11)	0.0232 (11)
C10B	0.0503 (14)	0.0494 (15)	0.0479 (15)	0.0201 (12)	0.0178 (12)	0.0321 (12)
C11A	0.0411 (13)	0.0319 (12)	0.0413 (14)	0.0061 (10)	0.0139 (11)	0.0198 (11)
C11B	0.0489 (14)	0.0394 (14)	0.0460 (15)	0.0121 (11)	0.0229 (12)	0.0221 (12)
C12A	0.0339 (12)	0.0365 (13)	0.0371 (13)	0.0058 (9)	0.0050 (10)	0.0155 (11)
C12B	0.0375 (12)	0.0401 (13)	0.0387 (13)	0.0111 (10)	0.0123 (10)	0.0177 (11)
C13A	0.0404 (13)	0.0379 (13)	0.0327 (13)	0.0104 (10)	0.0067 (10)	0.0191 (11)
C13B	0.0408 (13)	0.0402 (13)	0.0374 (13)	0.0136 (10)	0.0133 (10)	0.0229 (11)
N14A	0.0434 (12)	0.0460 (12)	0.0535 (14)	0.0072 (9)	0.0024 (10)	0.0315 (11)
N14B	0.0453 (12)	0.0751 (14)	0.0697 (16)	0.0203 (9)	0.0157 (11)	0.0506 (13)
N15A	0.0556 (14)	0.0400 (12)	0.0576 (14)	0.0050 (10)	0.0217 (11)	0.0249 (11)
N15B	0.0629 (15)	0.0514 (14)	0.0633 (16)	0.0033 (11)	0.0245 (13)	0.0306 (12)
N16A	0.0453 (12)	0.0518 (13)	0.0482 (13)	0.0064 (10)	0.0013 (10)	0.0328 (11)
N16B	0.0438 (12)	0.0560 (13)	0.0491 (13)	0.0166 (10)	0.0112 (10)	0.0311 (11)

### Geometric parameters (Å, °)

**Table:**

O1A—C8A	1.252 (2)	C5A—C6A	1.416 (3)
O1B—C8B	1.237 (3)	C5A—H5A	0.9500
O2A—N14A	1.214 (3)	C5B—C6B	1.393 (3)
O2B—N14B	1.208 (3)	C5B—H5B	0.9500
O3A—N14A	1.217 (2)	C6A—N7A	1.331 (3)
O3B—N14B	1.226 (2)	C6B—N7B	1.312 (3)
O4A—N15A	1.230 (3)	N7A—H7A	0.87 (3)
O4B—N15B	1.212 (3)	N7A—H7B	1.05 (3)
O5A—N15A	1.214 (3)	N7B—H7C	0.72 (3)
O5B—N15B	1.212 (3)	N7B—H7D	0.84 (3)
O6A—N16A	1.209 (2)	C8A—C13A	1.436 (3)
O6B—N16B	1.206 (2)	C8A—C9A	1.446 (3)
O7A—N16A	1.208 (2)	C8B—C13B	1.441 (3)
O7B—N16B	1.197 (2)	C8B—C9B	1.450 (3)
N1A—C6A	1.339 (3)	C9A—C10A	1.371 (3)
N1A—C2A	1.350 (3)	C9A—N14A	1.454 (3)
N1A—H1A	0.87 (3)	C9B—C10B	1.351 (3)
N1B—C6B	1.352 (3)	C9B—N14B	1.461 (3)
N1B—C2B	1.356 (3)	C10A—C11A	1.389 (3)
N1B—H1B	0.96 (3)	C10A—H10A	0.9500
C2A—C3A	1.348 (3)	C10B—C11B	1.381 (3)
C2A—H2A	0.9500	C10B—H10B	0.9500
C2B—C3B	1.347 (4)	C11A—C12A	1.367 (3)
C2B—H2B	0.9500	C11A—N15A	1.453 (3)
C3A—C4A	1.391 (4)	C11B—C12B	1.379 (3)
C3A—H3A	0.9500	C11B—N15B	1.457 (3)
C3B—C4B	1.390 (4)	C12A—C13A	1.374 (3)
C3B—H3B	0.9500	C12A—H12A	0.9500
C4A—C5A	1.348 (3)	C12B—C13B	1.359 (3)
C4A—H4A	0.9500	C12B—H12B	0.9500
C4B—C5B	1.351 (4)	C13A—N16A	1.456 (3)
C4B—H4B	0.9500	C13B—N16B	1.465 (3)
			
C6A—N1A—C2A	123.2 (2)	C10A—C9A—C8A	124.6 (2)
C6A—N1A—H1A	121.2 (17)	C10A—C9A—N14A	116.76 (19)
C2A—N1A—H1A	115.6 (17)	C8A—C9A—N14A	118.66 (19)
C6B—N1B—C2B	122.5 (2)	C10B—C9B—C8B	124.8 (2)
C6B—N1B—H1B	115.8 (15)	C10B—C9B—N14B	116.4 (2)
C2B—N1B—H1B	121.7 (15)	C8B—C9B—N14B	118.8 (2)
C3A—C2A—N1A	120.1 (3)	C9A—C10A—C11A	118.2 (2)
C3A—C2A—H2A	120.0	C9A—C10A—H10A	120.9
N1A—C2A—H2A	120.0	C11A—C10A—H10A	120.9
C3B—C2B—N1B	120.8 (3)	C9B—C10B—C11B	118.6 (2)
C3B—C2B—H2B	119.6	C9B—C10B—H10B	120.7
N1B—C2B—H2B	119.6	C11B—C10B—H10B	120.7
C2A—C3A—C4A	118.9 (2)	C12A—C11A—C10A	121.5 (2)
C2A—C3A—H3A	120.6	C12A—C11A—N15A	119.6 (2)
C4A—C3A—H3A	120.6	C10A—C11A—N15A	118.9 (2)
C2B—C3B—C4B	117.8 (3)	C12B—C11B—C10B	121.4 (2)
C2B—C3B—H3B	121.1	C12B—C11B—N15B	119.6 (2)
C4B—C3B—H3B	121.1	C10B—C11B—N15B	119.0 (2)
C5A—C4A—C3A	120.8 (2)	C11A—C12A—C13A	119.8 (2)
C5A—C4A—H4A	119.6	C11A—C12A—H12A	120.1
C3A—C4A—H4A	119.6	C13A—C12A—H12A	120.1
C5B—C4B—C3B	121.7 (2)	C13B—C12B—C11B	119.5 (2)
C5B—C4B—H4B	119.2	C13B—C12B—H12B	120.3
C3B—C4B—H4B	119.2	C11B—C12B—H12B	120.3
C4A—C5A—C6A	119.6 (3)	C12A—C13A—C8A	123.6 (2)
C4A—C5A—H5A	120.2	C12A—C13A—N16A	116.78 (19)
C6A—C5A—H5A	120.2	C8A—C13A—N16A	119.61 (19)
C4B—C5B—C6B	119.7 (2)	C12B—C13B—C8B	123.8 (2)
C4B—C5B—H5B	120.2	C12B—C13B—N16B	116.4 (2)
C6B—C5B—H5B	120.2	C8B—C13B—N16B	119.77 (19)
N7A—C6A—N1A	118.9 (2)	O2A—N14A—O3A	122.7 (2)
N7A—C6A—C5A	123.7 (2)	O2A—N14A—C9A	119.2 (2)
N1A—C6A—C5A	117.4 (2)	O3A—N14A—C9A	118.1 (2)
N7B—C6B—N1B	118.1 (2)	O2B—N14B—O3B	121.8 (2)
N7B—C6B—C5B	124.3 (2)	O2B—N14B—C9B	120.4 (2)
N1B—C6B—C5B	117.6 (2)	O3B—N14B—C9B	117.7 (2)
C6A—N7A—H7A	118 (2)	O5A—N15A—O4A	123.5 (2)
C6A—N7A—H7B	120.8 (17)	O5A—N15A—C11A	118.3 (2)
H7A—N7A—H7B	120 (3)	O4A—N15A—C11A	118.2 (2)
C6B—N7B—H7C	114 (2)	O4B—N15B—O5B	123.4 (2)
C6B—N7B—H7D	117.1 (19)	O4B—N15B—C11B	118.7 (2)
H7C—N7B—H7D	129 (3)	O5B—N15B—C11B	117.9 (2)
O1A—C8A—C13A	125.4 (2)	O7A—N16A—O6A	121.4 (2)
O1A—C8A—C9A	122.2 (2)	O7A—N16A—C13A	120.0 (2)
C13A—C8A—C9A	112.30 (19)	O6A—N16A—C13A	118.5 (2)
O1B—C8B—C13B	124.9 (2)	O7B—N16B—O6B	121.4 (2)
O1B—C8B—C9B	123.2 (2)	O7B—N16B—C13B	120.5 (2)
C13B—C8B—C9B	111.9 (2)	O6B—N16B—C13B	118.1 (2)

### Hydrogen-bond geometry (Å, °)

**Table:**

*D*—H···*A*	*D*—H	H···*A*	*D*···*A*	*D*—H···*A*
N1B—H1B···O1B	0.96 (3)	1.79 (3)	2.681 (3)	152 (2)
N1B—H1B···O7B	0.96 (3)	2.36 (3)	3.035 (3)	127 (2)
N7B—H7D···O1B	0.84 (3)	2.04 (3)	2.784 (3)	148 (3)
N7B—H7D···O2B	0.84 (3)	2.47 (3)	3.165 (3)	141 (2)
N1A—H1A···O1A	0.87 (3)	1.97 (3)	2.726 (2)	145 (2)
N1A—H1A···O7A	0.87 (3)	2.34 (3)	3.031 (3)	137 (2)
N7A—H7B···O2A	1.05 (3)	2.40 (3)	3.329 (3)	147 (2)
C10A—H10A···O4B^i^	0.95	2.57	3.438 (3)	153
N7A—H7A···O2B^ii^	0.87 (3)	2.24 (3)	3.029 (3)	152 (3)
N7B—H7C···O2A^ii^	0.72 (3)	2.49 (3)	3.137 (3)	149 (3)

Symmetry codes:  (i) −*x*, −*y*, −*z*+1; (ii) −*x*+1, −*y*+1, −*z*+1.
